# Supplementary material for: Transcriptome Profiles Reveal Key Regulatory Networks during Single and Multifactorial Stresses Coupled with Melatonin Treatment in Pitaya (Selenicereus undatus L.)
Source: Int J Mol Sci. 2024 Aug 15;25(16):8901. doi: 10.3390/ijms25168901 (PMC11354645; doi:10.3390/ijms25168901)
Supplement: Supplementary file 1 [file ijms-25-08901-s001.zip › Supplementary file S1 Table S1.pdf]

Supplementary Table S1. Sequencing data quality assessment.

Sample	Clean Reads Pairs	Clean base(bp)	Length	Q20(%)	Q30(%)	GC(%)
CdDM-1	22,522,062	6,756,618,600	150;150	98.52;96.47	95.10;89.58	46.37;46.35
CdDM-2	24,447,060	7,334,118,000	150;150	98.50;96.31	95.05;89.06	45.62;45.59
CdDSM-1	22,488,787	6,746,636,100	150;150	98.50;96.84	95.09;90.67	45.44;45.47
CdDSM-2	22,516,879	6,755,063,700	150;150	98.50;96.66	95.07;90.15	45.40;45.37
CK-1	23,250,968	6,975,290,400	150;150	98.64;96.56	95.46;89.88	45.68;45.67
CK-2	25,910,677	7,773,203,100	150;150	98.57;96.80	95.26;90.57	45.62;45.62
M-1	23,588,850	7,076,655,000	150;150	98.57;96.57	95.26;89.89	45.66;45.66
M-2	24,989,564	7,496,869,200	150;150	98.62;96.62	95.40;90.08	45.91;45.94
S-1	28,033,296	8,409,988,800	150;150	98.68;96.66	95.58;90.19	46.07;46.10
S-2	24,462,102	7,338,630,600	150;150	98.61;96.63	95.37;90.11	46.25;46.29
D-1	25,668,619	7,700,585,700	150;150	98.64;96.43	95.45;89.51	45.64;45.63
D-2	20,707,291	6,212,187,300	150;150	98.52;96.26	95.07;89.04	44.79;44.83
Cd-1	23,205,681	6,961,704,300	150;150	98.64;96.62	95.46;90.07	45.35;45.38
Cd-2	19,086,388	5,725,916,400	150;150	98.45;96.75	94.94;90.38	45.01;45.02
CdS-1	28,574,840	8,572,452,000	150;150	98.60;96.52	95.34;89.77	45.48;45.51
CdS-2	26,046,579	7,813,973,700	150;150	98.46;96.26	94.90;88.94	45.85;45.87
CdD-1	23,892,328	7,167,698,400	150;150	98.66;96.60	95.53;90.02	45.67;45.68
CdD-2	19,136,541	5,740,962,300	150;150	98.44;96.05	94.85;88.37	45.81;45.79
CdSD-1	28,614,210	8,584,263,000	150;150	98.63;96.75	95.42;90.44	45.74;45.77
CdSD-2	28,481,409	8,544,422,700	150;150	98.68;96.69	95.59;90.26	45.08;45.09
CdSM-1	30,788,568	9,236,570,400	150;150	98.66;96.77	95.53;90.50	45.43;45.44
CdSM-2	28,793,799	8,638,139,700	150;150	98.62;96.51	95.40;89.75	45.80;45.82
