# Supplementary material for: Transcriptome Profiles Reveal Key Regulatory Networks during Single and Multifactorial Stresses Coupled with Melatonin Treatment in Pitaya (Selenicereus undatus L.)
Source: Int J Mol Sci. 2024 Aug 15;25(16):8901. doi: 10.3390/ijms25168901 (PMC11354645; doi:10.3390/ijms25168901)
Supplement: Supplementary file 1 [file ijms-25-08901-s001.zip › Supplementrary file S2, Figure S1,S2,S3 and S4.pdf]

Up-regulated DEG GO enrichment

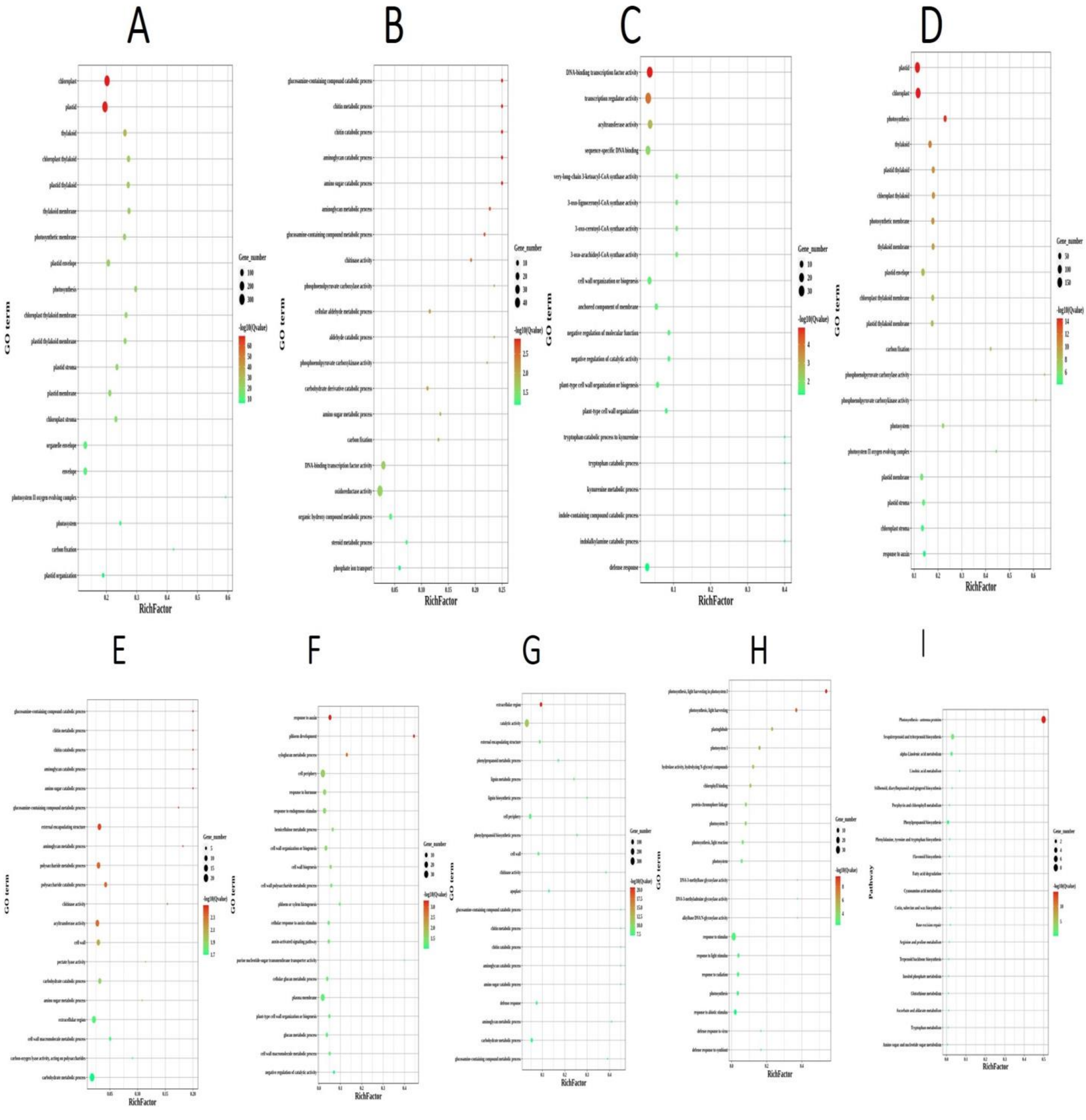

Fig S1. Top 20 GO enrichment of uP-regulated DEGs, (A) M-vs-Ck, (B) S-vs-CK, (C) D-vs-Ck, (D) Cd-vs-Ck, (E) CdD-vs-Ck, (F) CdS-vs-Ck, (G) CdS-vs-CdSM, (H) CdD-CdDM, (I) CdSD-vs-CdDSM

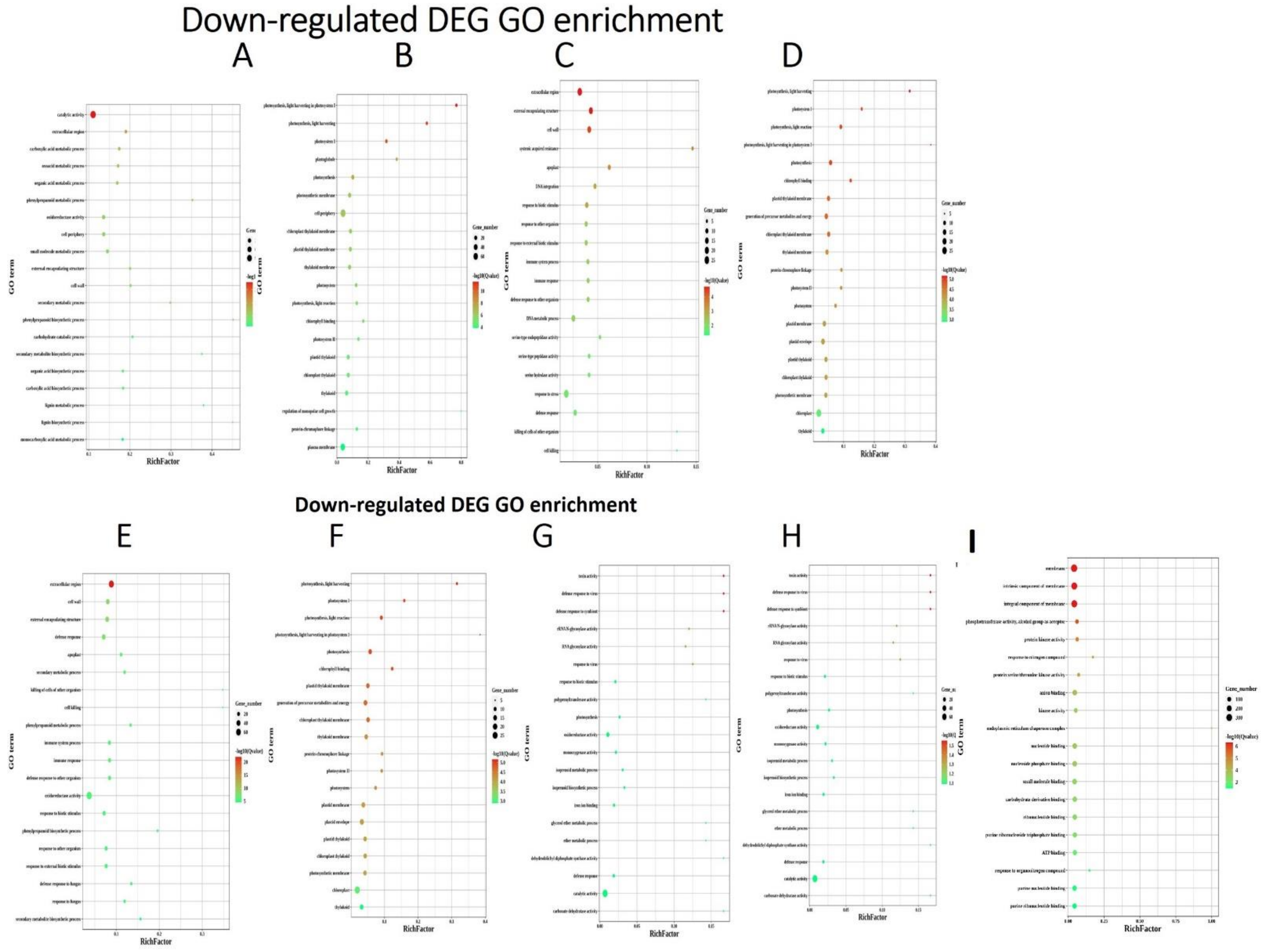

Fig S2. Top 20 GO enrichment of Down-regulated DEGs, (A) M-vs-Ck, (B) S-vs-CK, (C) D-vs-Ck, (D) Cd-vs-Ck, (E) CdD-vs-Ck, (F) CdS-vs-Ck, (G) CdS-vs-CdSM, (H) CdD-CdDM, (I) CdSD-vs-CdDSM

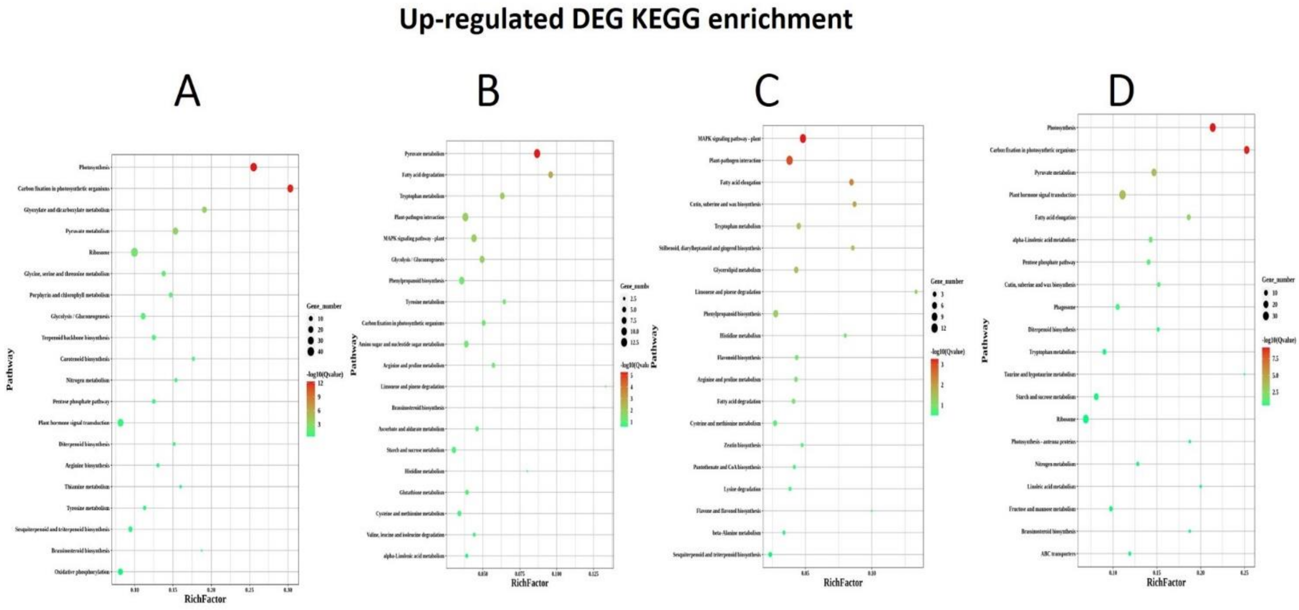

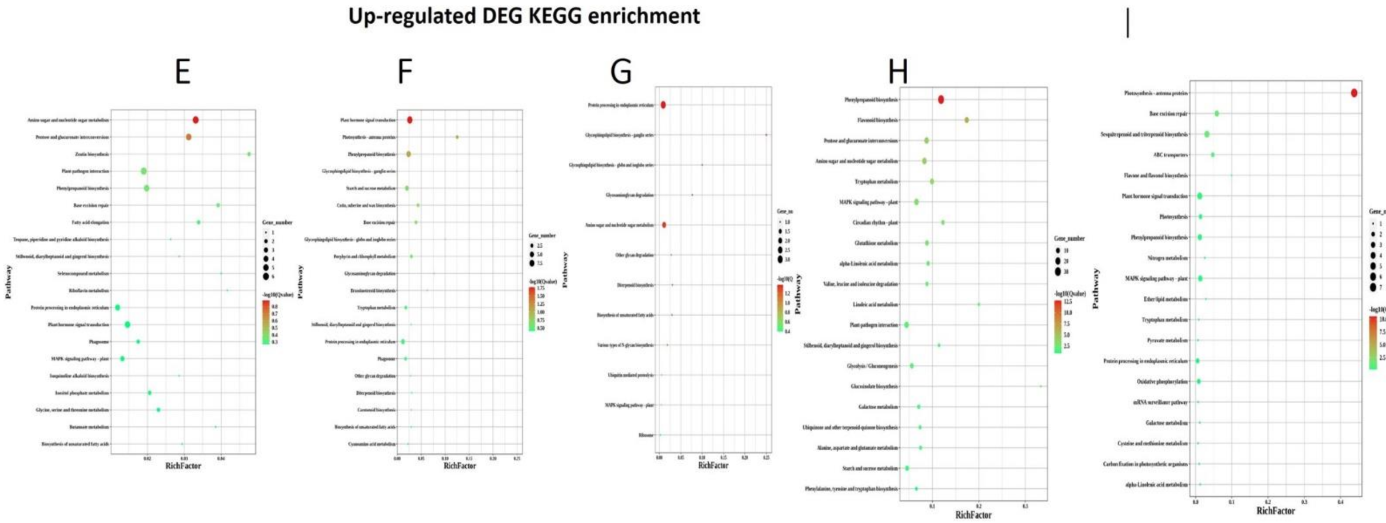

Fig S3. Top 20 KEGG enrichment of Up-regulated DEGs, (A) M-vs-Ck, (B) S-vs-CK, (C) D-vs-Ck, (D) Cd-vs-Ck, (E) CdD-vs-Ck, (F) CdS-vs-Ck, (G) CdS-vs-CdSM, (H) CdD-CdDM, (I) CdSD-vs-CdDSM

Down-regulated DEGs KEGG enrichments

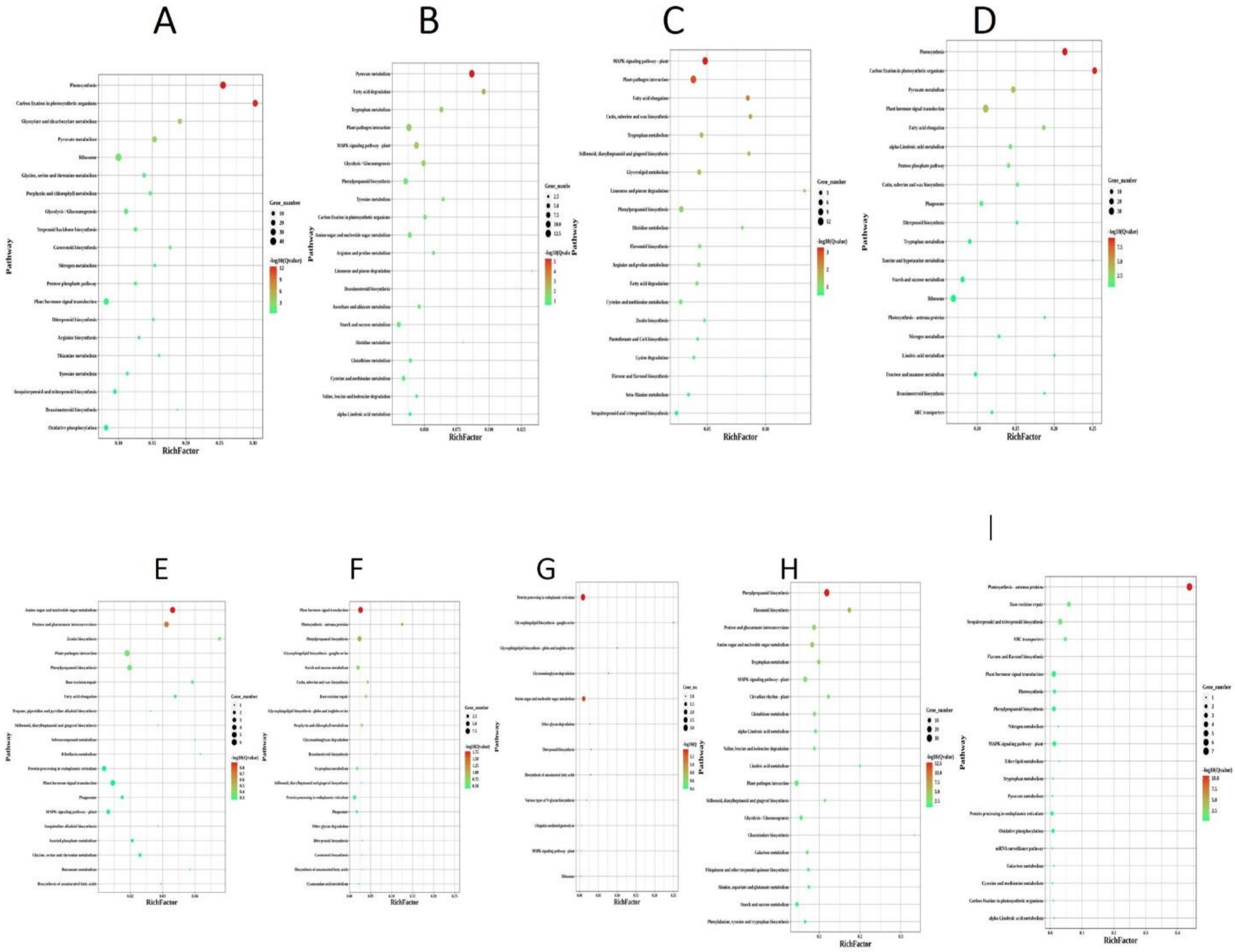

Fig S4. Top 20 KEGG enrichment of Down-regulated DEGs, (A) M-vs-Ck, (B) S-vs-CK, (C) D-vs-Ck, (D) Cd-vs-Ck, (E) CdD-vs-Ck, (F) CdS-vs-Ck, (G) CdS-vs-CdSM, (H) CdD-CdDM, (I) CdSD-vs-CdDSM
